# The role of endoscopic dilation and stents in refractory benign esophageal strictures: a retrospective analysis

**DOI:** 10.1186/s12876-019-1006-0

**Published:** 2019-06-20

**Authors:** Qing Lu, Hailin Yan, Yilan Wang, Tiantian Lei, Linlin Zhu, Hongsheng Ma, Jinlin Yang

**Affiliations:** 1 Department of Gastroenterology, West China Hospital of Sichuan University, No.37 Guo Xue Alley, Chengdu, 610041 Sichuan Province China; 2 Department of Day Surgery, West China hospital of Sichuan University, 37 Guoxue Road, Chengdu, 610041 Sichuan China

**Keywords:** Refractory benign esophageal strictures, Esophageal dilation, Esophageal stents, Long-term outcomes, Retrospective analysis

## Abstract

**Background:**

The outcomes of patients with refractory benign esophageal strictures (RBES) are unclear, and the clinical efficacy of dilation versus stent placement is lacking. Our objective was to explore the role of endoscopic dilation and stents placement in the management of RBES.

**Methods:**

RBES patients treated with dilation and stents in our hospital between January 2009 and December 2017 were included in this study. The primary outcomes were to assess clinical effectiveness and adverse events. The secondary outcome was to identify factors that predicted the dysphagia-free period.

**Results:**

Among 75 RBES patients (54 male; median age 59 years), 39 (52%), 20 (26.7%), 3 (4%), 10 (13.3%), and 3 (4%), were postsurgical, post-ESD, achalasia of cardia, caustic and mixed etiology, respectively. The median number of endoscopic therapy was 5 times (range 3, 21). Endoscopic therapy was successful in 46 patients (61.3%). Patients treated with dilation showed a higher success rate (70.9%, 39/55) than that treated with stents (35%, 7/20). Fifteen patients died during follow-up. Nineteen patients had adverse events after endoscopic therapy. In total, the mean dysphagia-free period was 3.4 months (95% CI, 2.5–4.3). The patients treated with dilation demonstrated a dysphagia-free period of 3.7 months (95% CI, 2.7–5), while patients treated with stents displayed a dysphagia-free period of 2.3 months (95% CI, 1.5–3). The dysphagia-free period had a linear growth trend over time, with an increase of 12 days per endoscopic therapy.

**Conclusion:**

The dysphagia-free period increased by 12 days per endoscopic therapy, so the endoscopic therapy tended to be effective in patients with RBES by increasing the dysphagia-free period. However, compared to dilation therapy, stent therapy was not effective in increasing the dysphasia-free period and reducing the times and frequency of dilation. In addition, univariate and multivariate analyses also indicated that etiology may predict the endoscopic therapy outcome.

**Trial registration:**

This study was retrospectively registered and approved by the Ethics Committee of West China Hospital of Sichuan University (IRB number: ChiCTR1800016321).

## Background

Benign strictures of the esophagus are presumed to be a sequela of deep esophageal injury, which stimulates production of fibrous tissue and deposition of collagen, in the absence of endoscopic or pathological evidence of malignant strictures. Benign strictures of the esophagus include peptic esophageal strictures caused by long-term acid reflux (reflux esophagitis), caustic esophageal strictures, postsurgical esophageal strictures, post-endoscopic submucosal dissection (ESD) esophageal strictures, strictures caused by local radiotherapy, or tuberculosis [1, 2]. The management of patients with benign esophageal stricture is time-consuming and challenging. Dilation with bougies or balloons is the classic treatment for esophageal strictures [3], but over 30% of patients need continuing endoscopic dilation for more than 2 sessions during long-term follow-up [4, 5]. Kochman defined RBES as more than 3–5 dilations having been performed without clinical and endoscopic response or when it was impossible to achieve a 14 mm lumen over 3 dilation sessions [6, 7]. Some studies suggest temporary placement of self-expandable stents for RBES when dilation has failed, though a clear definition of clinical failure has not been uniformly adopted. Stents were left to remodel the scar tissue. RBES patients with stents could alleviate symptoms of dysphagia and reduce the frequency of dilation [8, 9]. Current research confirms that both dilation and stents for RBES are effective, but literature is scarce on the clinical efficacy of dilation versus stents. In addition, the long-term outcomes of RBES remain unclear. In our study, patients were followed for 6 months to assess the long-term outcomes, safety, and efficacy of endoscopic therapy in patients with RBES after at least 3 endoscopic therapy sessions.

## METHODS

### Patients

**Fig. 1:**
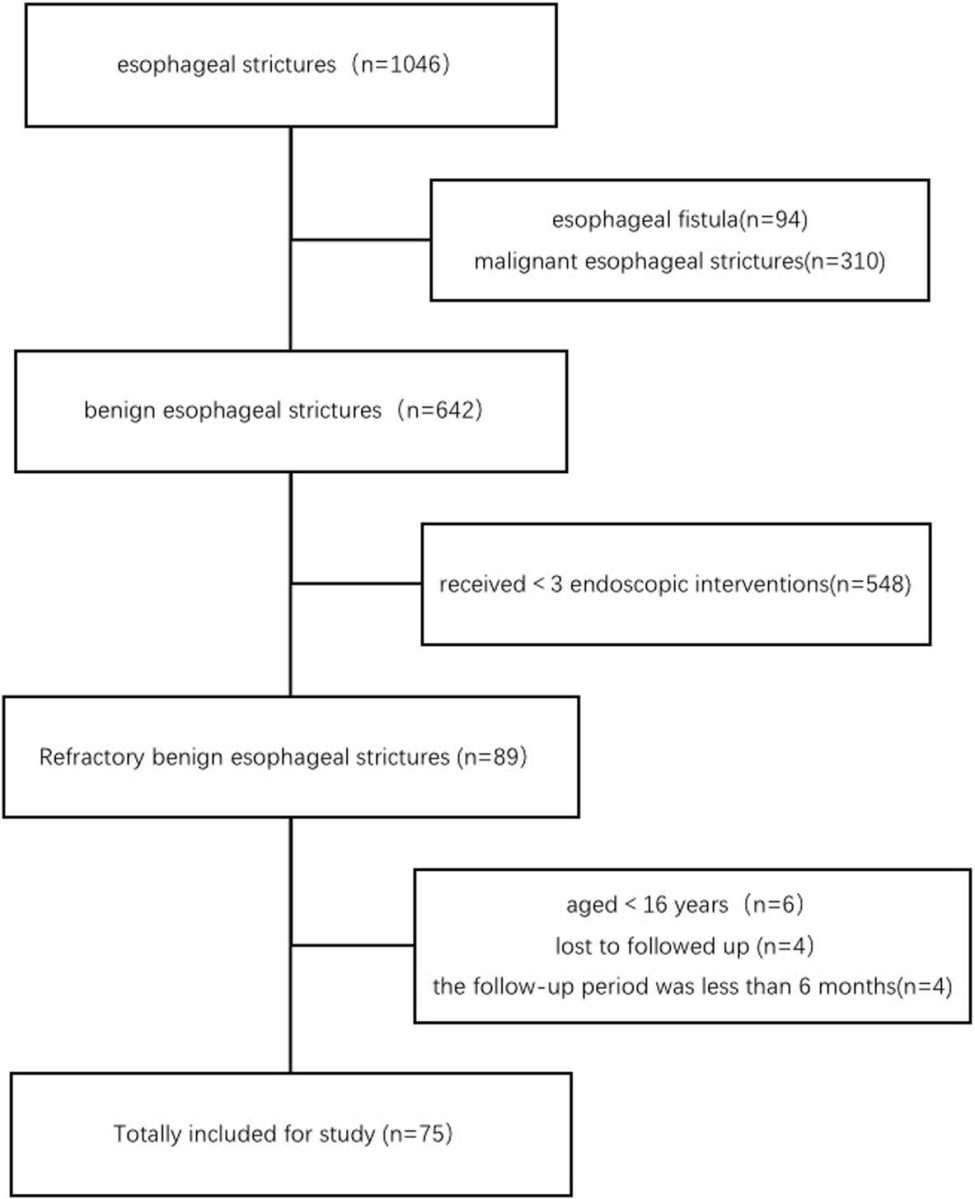
Flowchart of patients with RBES

This is a single-center retrospective study. One thousand forty-six patients with esophageal strictures were treated by dilation or stents between January 2009 and December 2017 in our hospital, and we excluded the patients with endoscopic incision. All medical records were retrospectively reviewed. Patients were included in the study if (1) they had been diagnosed with esophageal benign stricture according to clinical manifestation, gastroscopy, and pathology, (2) they had persistent symptoms of dysphagia with at least three sessions of endoscopic therapy, and (3) they had complete medical records and follow-up information, including gender, age at first therapy, RBES etiology, number, location, length and diameter of strictures, endoscopic therapy, the period between two consecutive endoscopic interventions, and adverse events. Patients were excluded if they (1) were younger than 16 years old, (2) had received less than 3 endoscopic interventions, (3) had been diagnosed with congenital esophageal stenosis, malignant esophageal strictures, or with esophageal fistula (Fig. 1). This study was approved by the Ethics Committee of West China Hospital of Sichuan University (IRB number: ChiCTR1800016321).

### Equipment and procedure

Patients were treated following local clinical practice in accordance with best practices. Gastroscopes (JIF - Q260 or JIF - Q260J, Olympus Medical Systems, Tokyo, Japan) were used according to the stricture characteristics and type of treatment. Each physician had over 10 years’ experience in endoscopy. Written consent for endoscopic dilation and stent was obtained before the operations. All patients received the standard dilation, by balloons and bougies or fully covered self-expandable metal stents (SEMSs) (MTN-SE C-membrane; 60–140 m; 20 mm; MicroTech, Nanjing, China). The dilation procedures were performed with a through-the-scope balloon (Type 5842; 3 ATM-12 mm, 4.5 ATM-13.5 mm, 8 ATM-15 mm; Boston Scientific Corp; Marlborough, America) and savary bougies system (Savary-Gilliard Dilator set, type SGD-70-1; length: 70 cm, diameters: 5 mm, 7 mm, 9 mm, 11 mm, 12.8 mm, 14 mm, 15 mm; Wilson-Cook Medical, America). Dilation was the standard treatment for all patients, with other alternatives, such as stents placed on a case-by-case basis at the discretion of the endoscopist performing the procedure, after an appropriate discussion with surgeons and a dietician, as needed. Patients with RBES undergoing esophageal dilation required multiple sessions, every 2 to 4 weeks or longer. Some patients with persistent dysphagia symptoms were unable to achieve acceptable symptom relief and needed an intensive dilation schedule, (e.g. as frequent as every 2 to 3 days). However, in our study, we considered all subsequent endoscopic interventions within 7 days as one treatment cycle. In our study stent removal was after performed 6–8 weeks (range 4–10 weeks) later to allow scar tissue remodeling.

### Endpoints

The primary outcomes were to assess clinical effectiveness and adverse events. Endoscopic therapy success was defined as a persistent dysphagia-free period, with no need for any other interventions, lasting at least 6 months [10]. Treatment failure was defined as the need for any endoscopic intervention, including surgery [11], during the follow-up period. Adverse events included esophageal fistula, bleeding, stent dysfunction (stent overgrowth, stent migration and food impaction), endoscopy to readjust or remove stents, and intolerable chest pain. Early and late adverse events were defined as those occurring within 7 days and later than 7 days after stenting, respectively. The secondary outcome was to identify factors that affected the dysphagia-free period. A dysphagia-free period was defined as a time interval between two subsequent endoscopic interventions greater than 7 days. We considered all endoscopic interventions within 7 days as only one treatment cycle. All patients were followed via telephone contact from the third therapy session until study termination or patient death to assess symptom resolution.

### Statistical methods

Descriptive statistics were reported as median or mean and range for continuous variables. The chi-square test and the Fisher’s exact test were used for categorical variables, and the t test was used for continuous variables. We used logistic regression analysis to estimate the risk factors influencing treatment success. To explore the trend in dysphagia-free period length over time, we used hierarchical linear models. The medical data of patients with RBES were divided into two layers. The dilation times were the first layer of data (dysphagia-free period), and the characteristics of the patient, stricture and therapy were the second layer (gender, age, etiology, number, location and length of strictures, initial dysphagia-free period, and endoscopic therapy). The hierarchical linear models explored prognostic factors that were associated independently with a trend in the dysphagia-free period. All nonnormal data were transformed to normality. The dysphagia-free period analyses were performed using HLM Statistics version 6 (Scientific Software International Inc., Lincolnwood, IL), and all other statistical analyses were performed using SPSS Statistics version 22.0 (IBM Corp, Armonk, NY). *P* values < 0.05 were considered to indicate statistical significance.

## Results

### Subject baseline characteristics

Patient characteristics are shown in Table 1. In total, 75 RBES patients were included (54 male; median age, 59 years) (Fig. 1). Among these 75 patients, 39 (52%), 20 (26.7%), 10(13.3%), 3 (4%), 3 (4%) showed esophageal strictures due to surgery, ESD, caustic injury, mixed etiology, and achalasia of cardia, respectively. Achalasia of cardia was considered as benign stenosis according to the UK guidelines on oesophageal dilation [12]. The mixed etiology patients causing by caustic injury firstly, after esophageal anastomosis colon surgery, had persistent dysphasia after receiving several dilation sessions. Among the 75 cases, 9 (12%), 59 (78.7%), 4 (5.3%), 3 (4%) demonstrated esophageal strictures in the cervical esophagus, thoracic esophagus, ventral esophagus, and both cervical and thoracic esophagus, respectively. 56 (74.7%) patients had a single stricture, 14 (18.7%) had two strictures, and 5(6.6%) patients had three or more strictures. The median strictures length was 2 cm (0.5 cm, 10.0 cm), the median strictures diameter was 4 mm (1.0 mm, 8.5 mm), and the median strictures final diameter reached after each dilation session was 10 mm (5 mm, 15 mm). All 75 patients received repeated endoscopic dilations. The median number of endoscopic therapies was 5 (range 3, 21), and a mean of 1.9 (range 1, 6) SEMSs were placed in 20 (26.7%) patients. The mean time of stent placement was 29 days (range 7, 67). The median follow-up time was 31 months (range 6, 106).

**Table 1:** The characteristics of 75 patients with RBES and univariate analysis of endoscopic treatment success

Category		N (%)/M (range)	P^c^
Sex	Female	21 (28)	0.554
	Male	54 (72)	
Age (year)	–	59 (16, 75)	0.94
Etiology^a^	ESD/ESTD	20 (26.7)	0.040
	Surgery	39 (52)	
	Achalasia of cardia	3 (4)	
	Caustic	10 (13.3)	
	Mixed	3 (4)	
Location of strictures^a^	Cervical	9 (12)	0.872
	Thoracic	59 (78.7)	
	Ventral	4 (5.3)	
	Mixed^b^	3 (4)	
Number of strictures	One	56 (74.7)	0.201
	Two	14 (18.7)	
	Three or more	5 (6.6)	
Length of strictures (cm)	–	2 (0.5, 10)	0.923
Diameter of strictures (mm)	–	4 (1, 8.5)	0.240
Diameter of strictures after dilation (mm)	–	10 (5, 15)	0.756
Times	–	5 (3, 21)	<0.001
Dysphagia-free period	–	71 (15, 777)	0.585
Endoscopic therapy	Stents + Dilation	20 (26.7)	0.005
	Dilation only	55 (73.3)	

*RBES* Refractory benign esophageal strictures

^a^: Fisher’s exact test

^b^: The mixed etiology was the patient with RBES caused by caustic injury and esophageal anastomosis following colon surgery

^c^: P indicates a significant relationship between characteristics of patients and endoscopic therapy in univariate analysis

### Dysphagia-free period

Overall, the mean dysphagia-free period of all 75 patients was 3.4 months (95% CI, 2.5–4.3). The dysphagia-free periods in patients treated with dilation and stents were 3.7 months (95% CI, 2.7–5), and 2.3 months (95% CI, 1.5–3), respectively. To explore the trend in the dysphagia-free period over time, we used hierarchical linear models (Table 2). The result of the fixed effect intercept and slope was significant (*P* < 0.001). The dysphagia-free period had a linear growth trend over time, with an increase of 12 days per endoscopic therapy (95% CI, 8.7–16.7). The last dysphagia-free period was estimated to be 72 days (95% CI, 61.8–83.6). We included patient characteristics, therapy characteristics and stricture characteristics in the second level data to estimate prognostic factors associated independently with the trend in the dysphagia-free period. There was no significant correlation between the dysphagia-free period time trend and patient age, sex, etiology, stricture location, stricture number, stricture length, therapy, or initial dysphagia-free period. However, achalasia of cardia (*p* < 0.001, 95% CI, 97.5–210.9), postsurgical strictures (*p* = 0.046, 95% CI, − 4.4-47.2), stricture length more than 2 cm (*p* = 0.015, 95% CI, − 48- -5.6) and initial dysphagia-free period (p < 0.001, 95% CI, 36.3–68.3) tended to positively correlated with the intercept of the dysphagia-free period time-trend. The achalasia of cardia patients and postsurgical strictures with the stricture length less than 2 cm had longer dysphagia-free periods, compared with those patients with post-ESD strictures and stricture length more than 2 cm. Random-effects results showed that the variance component of the second data layer was large, due to the large variation among patients. The variance component, however, significantly decreased in the second level after excluding some stricture characteristics, especially stricture length, number and location. As such, compared with patients and therapy characteristics, stricture characteristics explain more of the variation in the time-trend of the dysphagia-free period.

**Table 2:** The time-trend of dysphagia-free period with RBES in the model of HLM

Fixed effects		Parameter (95% CI)	P
Times	Intercept (d)	72.7 (61.8–83.6)	<0.001
	Time-trend (d/time)	12.7 (8.7–16.7)	<0.001
Patient characteristics			
Sex			
Female–reference category	Intercept (d)	75.1 (54.3–95.9)	<0.001
	Time-trend (d/time)	10.5 (2.3–18.7)	0.014
Male	Intercept (d)	−3.2 (−27.8–21.4)	0.802
	Time-trend (d/time)	6.1 (−4.0–16.2)	0.236
Age			
	Intercept (d)	83.3 (46.4–120.2)	<0.001
	Time-trend (d/time)	23.6 (8.6–38.6)	<0.001
	Intercept (d)	−0.17 (−0.8–0.4)	0.557
	Time-trend (d/time)	−0.17 (− 0.4–0.1)	0.193
Stricture characteristics			
Etiology			
ESD/ESTD–reference category	Intercept (d)	57.6 (35.6–79.6)	< 0.001
	Time-trend (d/time)	14.7 (8.6–20.8)	0.001
Achalasia of cardia	Intercept (d)	154.2 (97.5–210.9)	< 0.001
	Time-trend (d/time)	−0.54 (−21.9–20.8)	0.971
Surgery	Intercept (d)	21.4 (−4.4–47.2)	0.046
	Time-trend (d/time)	9.01 (−6.7–24.7)	0.154
Caustic	Intercept (d)	−1.19 (−21.0–18.6)	0.931
	Time-trend (d/time)	−4.50 (−16.6–7.6)	0.575
Location			
	Intercept (d)	32.8 (7.1–58.5)	0.015
	Time-trend (d/time)	5.53 (−4.3–15.3)	0.273
	Intercept (d)	1.75 (0.7–2.8)	0.002
	Time-trend (d/time)	0.36 (−0.1–0.8)	0.101
Length			
<2 cm-reference category	Intercept (d)	87.7 (71.7–103.7)	<0.001
	Time-trend (d/time)	17.6 (9.4–25.8)	<0.001
≥2 cm	Intercept (d)	−26.8 (−48- -5.6)	0.015
	Time-trend (d/time)	−6.32 (−16.1–3.5)	0.212
Number			
One-reference category	Intercept (d)	79.9 (67.2–92.6)	<0.001
	Time-trend (d/time)	14.9 (9.8–20.0)	<0.001
Two or more	Intercept (d)	−24.8 (−48- -1.6)	0.040
	Time-trend (d/time)	−6.57 (−14.8–1.7)	0.123
Therapy characteristics			
Initial dysphagia-free period			
	Intercept (d)	52.3 (36.3–68.3)	<0.001
	Time-trend (d/time)	17.6 (10.0–25.2)	<0.001
	Intercept (d)	0.46 (0.2–0.7)	0.010
	Time-trend (d/time)	−0.12 (−0.3–0.1)	0.229
Therapy			
Dilation + Stents–reference category	Intercept (d)	74.9 (61.5–88.3)	<0.001
	Time-trend (d/time)	17.4 (11.1–23.7)	<0.001
Stents	Intercept (d)	−6.6 (−30.1–16.9)	0.583
	Time-trend (d/time)	−7.6 (−16.6–1.4)	0.103

*RBES* Refractory benign esophageal strictures

*CI* Confidence interval

*HLM* Hierarchical linear Model

### Clinical outcomes and adverse events

Endoscopic therapy was successful in 46 (61.3%) patients. The success rate of dilation treatment (70.9%, 39/55) was higher than that of stents (35%, 7/20). Endoscopic treatment failed in 29 (38.7%) patients. In 27 (36%) patients, the end of follow-up was coincident with the last endoscopic treatment. One (1.4%) patient was treated with surgery, and another (1.4%) patient was treated with endoscopic gastrostomy feeding tubes. Nineteen patients had adverse events after endoscopic therapy. Six (8%) patients experienced esophageal fistula, caused by ESD (1/75), surgery (1/75), caustic (2/75) and mixed etiology (2/75), respectively. Three (4%) patients experienced intolerable chest pain, and one (1.3%) patient experienced bleeding. Ten (13.3%) patients experienced stent dysfunction including stent overgrowth in 3 (4%), stent migration in 6 (8%) and food impaction in 5 (6.7%) (Table 3). Fifteen patients died during follow-up, including lung and stomach tumor metastasis, severe pulmonary infection, and other causes unrelated to any endoscopic procedure.

**Table 3:** Adverse events of RBES

	Early period no. (%)	Late periody no. (%)
	*N* = 5	*N* = 14
Stent overgrowth	0	3
Stent migration	0	6
Food impaction	0	5
Fistula	1	5
Bleeding	1	0
Intolerable chest pain	3	0

*RBES* Refractory benign esophageal strictures

Early and late adverse events: were defined as those occurring within 7 days and later than 7 days after stenting

There was a significant correlation between endoscopic treatment success and etiology, number of dilations and endoscopic therapy at univariate analysis (Table 1). In 39 patients with postsurgical stenosis, 18 patients had postoperative chemotherapy or radiotherapy. All the patients with a history of postoperative chemoradiation in our study had negative biopsies of the stricture. There was an insignificant correlation between therapy success and postoperative chemotherapy (95% CI, 0.109–1.814; *p* = 0.254) and postoperative radiotherapy (95% CI, 0.131–1.907; *p* = 0.307). Comparing bougies with balloons dilation, it showed no difference in terms of clinical resolution (*p* = 0.137). There was an insignificant correlation between the diameter after each dilatation session (*p* = 0.756) and the length of stenosis (*p* = 0.923) with endoscopic treatment success at univariate analysis. We included sex, age, etiology, dilation times, and location of strictures in the multivariate analysis. The multivariate analysis (Table 4) revealed that postsurgical strictures (OR, 13.082; 95% CI, 1.708–100.173; *P* = 0.013) and therapy times (OR, 1.714; 95% CI, 1.267–2.319; *P*<0.001) were the significant predictive factors for clinical resolution. The probability of endoscopic treatment failure was high in patients with postsurgical strictures. Both univariate and multivariate analyses suggested a correlation between etiology and clinical outcome. Therefore, we divided the etiology into four subgroups. The wide confidence interval of postsurgical strictures maybe caused by too many subgroups. It still had certain clinical significance. We conclude that the number of dilation was directly correlated with the probability of endoscopic treatment failure. Meanwhile, age (OR, 0.931; 95% CI, 0.868–0.998; *P* = 0.043) and caustic strictures (OR, 0.02; 95% CI, 0.001–0.418; *P* = 0.012) were related to clinical resolution. The treatment outcome tended to be better with age. But the statistical significance was weak. It hardly to say that there was clear clinical significance. Sex and other stricture characteristics were not significant predictive factors.

**Table 4:** Multivariate analysis of RBS

	Success no. (%)	P	OR (95% CI)
	*N* = 46		
Sex			
Male	32 (59.2%)	0.581	1.298 (0.514–3.276)
Female	14 (66.7%)	0.581	0.77 (0.305–1.944)
Age	–	0.043	0.931 (0.868–0.998)
Etiology			
Achalasia of cardia	2 (66.6%)	0.878	0.749 (0.019–30.15)
ESD/ESTD	10 (50%)	0.106	5.072 (0.707–36.363)
Surgery	22 (56.4%)	0.013	13.082 (1.708–100.173)
Caustic^a^	12 (92.3%)	0.012	0.02 (0.001–0.418)
Times	–	<0.001	1.714 (1.267–2.319)
Location of strictures^b^			
Upper (≤20)	12 (54.5%)	0.351	1.748 (0.540–5.659)
Middle (20<*n* ≤ 30)	25 (62.5%)	0.891	1.069 (0.413–2.764)
Distal (>30)	9 (69.2%)	0.392	0.535 (0.128–2.243)

*RBES* Refractory benign esophageal strictures

^a^: Caustic and Mixed

^b^: By endoscopy: Upper esophagus defined as ≤20 cm from the incisors, Middle esophagus 20 cm to 30 cm from the incisors, Distal esophagus>30 from the incisors

## Discussion

There is no universal definition for RBES, and the exact number of additional dilation sessions required before categorizing strictures as refractory varies among different series. In this study, we defined RBES as refractory to at least 3 endoscopic treatment sessions. One thousand forty-six patients with esophageal strictures were treated by dilation or stents between January 2009 and December 2017 in our hospital. Less than 10% population was the non-local population from other province, and the majority was the local population of Sichuan province. Almost all the Chinese people belong to the yellow race and same ethnic group. So the patients are very representative, and are considered as a homogeneous population. We established the database and selected patients with a medical record of nearly 8 years. Three-fifths of the patients achieved endoscopic therapy success, and nearly two-fifths of patients required constant endoscopic treatment before the end of the follow-up.

In our study, increased number of dilation was associated with a greater probability of endoscopic treatment failure. We considered that each round of dilation may entail multiple dilations, and patients with RBES may have negative results in long-term follow-up.

We also found that endoscopic therapy tended to increase the dysphagia-free period per dilation in patients with RBES. In most prior esophageal strictures researches, dysphagia score was often used as the endpoint without a consistent standard. However, in our study, the mean length of the dysphagia-free period was considered a proxy for survival quality. Our study showed the dysphagia-free period had a linear growth time trend, with an increase of 12 days per endoscopic therapy. We found no significant predictive factors for this growth rate.

The clinical outcomes of RBES relied on stricture features. The dysphagia-free period was more varied by features of stenosis as compared to the characteristics of patients and therapy. In addition, different stricture etiologies, with different mechanisms, may have different outcomes. Univariate and multivariate analyses explained that etiology may predict the endoscopic therapy outcomes. Postsurgical stricture etiology was a significant predictive factor for treatment failure in this study. The pathogenesis of anastomotic structures was due to reflux of corrosive gastric contents, ischemia of the residual tissue and inconsistencies of local hyperplasia [13, 14]. For post-ESD strictures, the residual ulcer surface may undergo self-repair, leading to fibrous overgrowth and scar contracture [15]. Therefore, we hypothesize that temporary endoscopic treatment was ineffective to change the depth of the esophageal wall and fiber formation. Caustic strictures were associated with a greater need for subsequent dilatations, due to that a long-lasting local inflammatory process leads to tissue fibrosis, transmural process and irreversible deposition of collagen [16]. In the multivariate analysis, caustic strictures were related to clinical resolution. However, we hypothesize caustic strictures tend to not have total clinical resolution after repeated endoscopy therapy, because of the longer dysphagia-free period (> 6 months) and subsequent late interventions. Among the caustic stricture patients, three patients underwent surgical intervention. For radiation-induced strictures, a high long-term recurrence rate of up to 33% has been shown, and 43% of these strictures were refractory to dilation therapy [17]. Post-radiotherapy strictures have been reported to account for 12% of benign strictures [18]. In our study, we did not separate postoperative chemoradiotherapy from postsurgical stenosis, but we found no statistical correlation between postsurgical chemoradiotherapy and clinical resolution.

It is difficult to say whether SMESs provide dysphagia relief in patients with RBES. Stents were left to remodel scar tissue, prevent stricture recurrence and improve dysphagia. Accordingly, some RBES patients experienced alleviation of dysphagia symptoms and reduced dilation times by stents [19]. However, this study showed that compared to dilation only, the dysphasia-free period of dilation with stents was not increased significantly. The result was same as the study of Repici. Repici concluded that once a stricture fits the definition of RBES, its prognosis relies on its being refractory, and endoscopic stenting does not affect the long-term natural history of the disease [20]. Stent therapy did not reduce the length and frequency of dilation.

On the other hand, the stents had more adverse events than dilation. A meta-analysis of eighteen studies (444 patients with RBES) conducted by Fuccio L et al. showed migration and adverse events rates of 28.6 and 20.6%, respectively [10, 21]. In our study, 31.6% patients (6/19) had migration, and 47.4% patients (9/19) had stent-related adverse events. Esophageal fistula is also a major adverse event, occurring in 0.1 to 0.3% patients after dilation [22]. In our study, 8% (6/75) of patients had an esophageal fistula, and the incidence was higher in caustic strictures patients (4/6). Caustic esophageal stenosis, with deep and wide esophageal lesions involving the whole esophageal wall, had a greater association with surgery, compared to repeated dilations, which may have low security and high risk of esophageal fistula [23]. The mean length of stenting was 29 days. It means the most patients had enough duration of stenting. Only 7 sessions of stenting were removed less than 29 days. The short duration of stenting was caused by the complication and poor physical condition, such as stent migration and intolerable chest pain needing removing. In our study, the stents had more adverse events than dilation. The short-term adverse event leading to worse outcome was another evidence, which confirmed that the SMESs could not provide dysphagia relief than dilation.

Surgery was performed in 1.3% (1/75) of patients, indicating that endoscopic dilation was considered as an optimal and long-term option for the management of patients with RBES. The majority of patients with RBES still required repeated dilation sessions.

This study has 3 limitations. First, our study had a nonrandomized design and potential selection bias, which inherently decreases the statistical power of the results. Second, our study was a retrospective single-center design with small sample size. Finally, we did not analyze the influence of different stent types on RBES patient outcomes. The recent randomized study from Walter concluded that biodegradable stent placement is associated with temporary reduction in number of repeat dilations and prolonged time to recurrent dysphagia compared with dilation [24]. In our study we did not analyze the different stent types, but current guidelines do not recommend a specific type of stents to be superior to any other. To better identify the factors that predict endoscopic therapy efficacy, adverse events, and dysphagia-free period, a large, prospective, randomized, and controlled study is needed.

## Conclusion

Overall, the dysphagia-free period increased by 12 days per endoscopic therapy, so the endoscopic dilation and stents tended to be effective in patients with RBES by increasing the dysphagia-free period. However, compared to dilation therapy, stent therapy was not effective in increasing the dysphasia-free period and reducing the times and frequency of dilation. The etiology of strictures may predict the endoscopic therapy outcome, and postsurgical strictures were a significant predictive factor for treatment failure.

## Data Availability

The datasets analysed during the current study are not publicly available, but are available from the corresponding author on reasonable request.

## Abbreviations

ESD: Endoscopic submucosal dissection
RBES: Refractory benign esophageal strictures
SEMSs: Self-expandable metal stents
